# High bone turnover assessed by 18F-fluoride PET/CT in the spine and sacroiliac joints of patients with ankylosing spondylitis: comparison with inflammatory lesions detected by whole body MRI

**DOI:** 10.1186/2191-219X-2-38

**Published:** 2012-07-12

**Authors:** Dorothee R Fischer, Christian W A Pfirrmann, Veronika Zubler, Katrin D M Stumpe, Burkhardt Seifert, Klaus Strobel, Giorgio Tamborrini, Gustav K von Schulthess, Beat A Michel, Adrian Ciurea

**Affiliations:** 1 Department of Nuclear Medicine, University Hospital Zurich, Raemistrasse 100, Zurich, CH-8091, Switzerland; 2 Department of Radiology, Orthopedic University Hospital Balgrist, Forchstrasse 340, Zurich, CH-8008, Switzerland; 3 Division of Biostatistics, Institute for Social and Preventive Medicine, University of Zurich, Hirschengraben 84, Zurich, CH-8001, Switzerland; 4 Department of Rheumatology, University Hospital Zurich, Raemistrasse 100, Zurich, CH-8091, Switzerland

**Keywords:** 18F-fluoride PET/CT, Whole-body MRI, Ankylosing spondylitis, Syndesmophytes, Inflammation

## Abstract

**Background:**

This study compares the frequency and distribution of increased activity on 18 F-fluoride PET/CT with the presence of bone marrow edema on whole-body MR imaging in the spine and sacroiliac joints (SIJ) of patients with active ankylosing spondylitis (AS).

**Methods:**

Ten patients (6 men and 4 women), between 30 and 58 years old (median 44) with active AS, were prospectively examined with both whole-body MRI and 18 F-fluoride PET/CT. Patients fulfilled modified NY criteria and had a Bath Ankylosing Spondylitis Disease Activity Index (BASDAI) of at least 4. Increased radiotracer uptake in PET/CT and bone marrow edema in whole-body MRI of spine and SIJ was evaluated independently by two blinded observers for each modality. Kappa statistics were used to compare interobserver agreement as well as scores of consensus reading of the two imaging modalities.

**Results:**

Analysis of interobserver agreement for PET/CT yielded a kappa value of 0.68 for spinal lesions and of 0.88 for SIJ lesions. The corresponding kappa values for the MRI modality were 0.64 and 0.93, respectively. More spinal lesions were detected by MRI in comparison to PET/CT (68 vs. 38), whereas a similar number of SIJ quadrants scored positive in both modalities (19 vs. 17). Analysis of agreement of lesion detection between both imaging modalities yielded a kappa value of only 0.25 for spinal lesions and of 0.64 for SIJ lesions.

**Conclusion:**

Increased 18 F-fluoride uptake in PET/CT is only modestly associated with bone marrow edema on MRI in the spine and SIJ of patients with AS, suggesting different aspects of bone involvement in AS.

## Background

Ankylosing spondylitis (AS) is the main subtype of a group of chronic inflammatory rheumatic diseases, called the spondyloarthritides (SpA), which mainly affect the axial skeleton as well as the peripheral joints and entheses [1-3]. The main clinical manifestations, inflammatory back pain and stiffness, are caused by inflammatory changes in the sacroiliac joints (SIJ) and spinal structures (sacroiliitis, spondylitis, spondylodiscitis, spondyloarthritis, and enthesitis). The clinical symptoms are paralleled over time by some osteodestructive and typical osteoproliferative changes of SIJ and spine (erosions, syndesmophytes, and ankylosis) best assessed by conventional radiographs [4]. MRI is able to detect acute inflammation of the SIJ and the spine before structural changes develop and has become a pivotal imaging tool for early diagnosis of axial SpA (recently included as a major criterion in the ASAS classification criteria) [5,6]. It has also allowed insights into the development of new bone from prior inflammatory lesions. The current paradigm involves resolution of an inflammatory lesion and its replacement by fat tissue followed by cartilage metaplasia and osteoproliferation through endochondral ossification [7,8]. This is exemplified by the observation that syndesmophytes seem to preferentially develop at fatty degenerated vertebral corners after resolution of inflammation [9,10]. However, syndesmophytes may also develop at sites without inflammation on baseline and follow up MRI, potentially through noninflammatory pathways [11,12].

High bone turnover assessed by nuclear medicine methods may detect osteoproliferative processes regardless of the inflammatory or noninflammatory origin [13]. While exact localization of regions of radionuclide uptake is difficult with conventional scintigraphy, 18F-fluoride PET/CT (F PET/CT) has a much better spatial resolution and sensitivity [14,15]. In the present pilot study, we systematically assessed regions of high bone turnover as detected by F PET/CT within the spine and the SIJ. We compared the distribution of the lesions with the inflammation detected as bone marrow edema on short tau inversion recovery (STIR) sequences by whole-body MRI in the same patients.

## Methods

### Patients

#### Study population

Ten patients with active AS were prospectively recruited in the Department of Rheumatology at the University Hospital Zurich from a national population-based prospective observational registry of axial spondyloarthritis patients (Swiss Clinical Quality Management (SCQM) in axial spondyloarthritis) and examined with both whole-body MRI and 18 F-fluoride PET/CT. We received approval from our Institutional Review Board (IRB) as well as a written informed consent of each patient.

#### Inclusion criteria

Patients were included if they fulfilled the modified NY criteria for AS and had a Bath Ankylosing Spondylitis Disease Activity Index (BASDAI) of at least 4.

#### Exclusion criteria

Exclusion criteria were ongoing or previous therapy (within the preceding 6 months) with tumor necrosis factor alpha inhibitors, pregnancy, contraindications for MRI imaging including cardiac pacemakers and neurostimulators, prior spine surgery/metal implants, as well as a time interval between F PET/CT and MRI of more than 6 weeks.

### Imaging protocol

#### Fluoride PET/CT scanning

All patients were examined in supine position on a combined PET/CT system (Discovery RX or Discovery STE, GE Healthcare, Milwaukee, WI, USA). These in-line systems contain a PET scanner and a multi-slice helical CT scanner (16 or 64 slices) permitting the acquisition of co-registered CT and PET images in the same session. Scanning was started 30 to 45 min after the i.v. injection of a median dose of 152 MBq (range 107 to 200 MBq) of 18 F-fluoride. Directly after the low-dose CT acquisition (FOV 50 cm, 30 to 80 auto mA, 140 kV, slice-thickness 3.75 mm, 0.5-s rotation time, standard reconstruction type), 3D-PET emission data were acquired for 1.5 and 2 min/cradle position, respectively. The CT data were used for attenuation correction as well as for the determination of anatomic distribution of increased radionuclide uptake, and images were reconstructed using a fully 3D iterative algorithm (VUE Point HD). Postprocessing of the acquired images was performed with a dedicated software (Volume Viewer PET/CT, AW 4.4 workstation, GE Healthcare) providing multiplanar reformatted images of PET alone, CT alone, and fused PET/CT.

#### MRI protocol

Whole-body MR imaging was performed in all patients using coronal and sagittal T1-weighted and STIR sequences of the entire spine and sacrum, anterior chest wall and shoulder, and pelvis. MR imaging was performed on a 1.5-T magnet (Siemens Medical Solutions, Erlangen, Germany), equipped with 18 independent radiofrequency channels. A body matrix coil was used. Coronal T1-weighted spin-echo (repetition time (TR), 573 ms; echo time (TE), 12 ms; PAT Factor 2; PAT mode GRAPPA (generalized autocalibrating partially parallel acquisition)) and coronal turbo STIR (TR, 9,860 ms; TE, 99 ms; TI, 130 ms; Turbo Factor 21, PAT Factor 2; PAT mode GRAPPA) sequences were acquired using two imaging steps with a FOV of 450 mm × 450 mm and an imaging matrix of 348 × 267 pixels/step, 5-mm slice thickness, interslice gap 1 mm, 32 sections resulting in a FOV of 785 × 450 mm. Sagittal T1-weighted spin-echo (TR, 403 ms; TE, 11 ms; PAT Factor 2; PAT mode GRAPPA) and sagittal turbo STIR (TR, 6,270 ms; TE, 93 ms; TI, 130 ms; Turbo Factor 21, PAT Factor 2, PAT mode GRAPPA) sequences were acquired using two imaging steps with a FOV of 450 mm × 450 mm and an imaging matrix of 448 × 268 pixels/step, 3-mm slice thickness, interslice gap 0.3 mm, 20 sections resulting in a FOV of 792 × 450 mm. Additional scans from the SI joint centers included coronal T1-weighted turbo spin-echo and STIR sequences angled parallel to the SI joint, and a transverse STIR sequence perpendicular to the coronal sequences. The scan parameters for the two coronal sequences were 19 slices, 4-mm slice thickness, 0.4-mm interslice gap, and field of view of 280 mm. For the coronal T1-weighted sequence, TR was 423 to 450 msec, TE was 12 to 13 msec, echo train length (ETL) was 3, and matrix was 512 × 256 pixels. For the coronal STIR sequence, the values were TR 3,700 to 4,930 msec, inversion time 145 to 150 msec, TE 50 to 69 msec, ETL 7 to 9, and matrix 256 to 384 × 256 pixels. For the transverse sequence, the values were 19 slices, 4.5-mm slice thickness, 0.6-mm interslice gap, field of view of 280 mm, TR was 4,930 msec, inversion time was 150 msec, TE was 69 msec, ETL was 9, and matrix was 256 × 256 pixels.

#### Analysis of MRI

Scoring of MR images was done by two readers (CWAP and VZ; staff radiologists experienced in musculoskeletal MRI) not involved in patient recruitment and blinded to clinical parameters and fluoride PET/CT. Increased bone marrow signal, denoting inflammation in the SIJ, vertebral bodies, and posterior spine elements was assessed on STIR sequences. Positive T2 signal for inflammation was defined as a signal that is greater than the signal from the center of an adjacent normal vertebral body or bone.

#### Analysis of 18 F-fluoride PET/CT

Two nuclear physicians (DRF and KDMS) not involved in patient recruitment and blinded to clinical parameters, and MRI analyzed PET/CT images visually. Increased 18 F-fluoride activity, as a surrogate marker of inflammation in the SIJ, vertebral bodies, and posterior spine elements, was assessed on PET/CT. Increased activity was defined as an activity that is greater than the activity in the center of an adjacent normal vertebral body or bone.

### Reading

Reading of MRI or PET/CT was first done separately to evaluate interobserver agreement. Images were then read in consensus. Increased radionuclide uptake on PET/CT was assessed in sagittal, axial, and coronal planes. Bone marrow edema on MRI was assessed in coronal and sagittal planes for the spine and in coronal and axial planes for the SIJ.

### Spinal lesions

#### Spine

The following specific lesions were recorded in a dichotomous manner (present/absent): vertebral corner inflammatory lesions (CIL; anterior (a) and posterior (p), upper (u) or lower (l)), vertebral lateral inflammatory lesions (LIL; right (ri) or left (le); upper (u) or lower (l)), vertebral non-corner inflammatory lesions (NIL), and two components of the posterior structures: first the posterior elements (posterior element inflammatory lesion (PIL)) including transverse and spinous process, costovertebral and costotransversal joints, and secondly the facet joints (facet joint inflammatory lesion (FIL)). In the cervical spine, as it is not possible to apply these definitions because the individual components of the posterior elements cannot be reliably separated, the presence of abnormal T2 signal or PET activity in any posterior element of the cervical spine was recorded dichotomously, treating the posterior arch of the vertebra as a single structure (cervical posterior inflammatory lesion (CPIL)) [16,17].

The following attribution rules were applied to avoid double counting and to assist with agreement analysis:

1. Bilateral lesions for FIL and more than one lesion for NIL, PIL, and CPIL were counted as a single positive for that structure.

2. Lesions in facet joints were assigned to the vertebral levels of the inferior facet of the joint.

3. CIL were assessed on sagittal images depicting the spinal channel; LIL were assessed on sagittal images strictly lateral to the spinal channel.

### SIJ lesions

Each SI joint was scored in four quadrants (superior and inferior iliac and sacral quadrants) regardless of the extent of the lesion (orientation strictly coronal to the body axis).

As a last step, all lesions that had revealed increased tracer uptake on PET/CT and no edema on MRI in consensus reading were again analyzed by one reader (VZ) with regard to the presence of fatty alterations on T1-weighted MRI sequences at these locations.

### Statistics

Agreement of detection of lesions between the readers and modalities was analyzed using kappa values. At first, kappa values were calculated regarding the agreement of detection between readers in PET/CT (DRF and KDMS) and in MRI (CWAP and VZ) separately for spinal and SIJ lesions. As a next step, consensus reading of PET/CT and MRI was analyzed calculating kappa values for the agreement of lesion detection between both imaging modalities again separately for spinal and SIJ lesions. Because of the assessment of several localizations within the same patient and the small number of patients, no confidence limits are presented. Statistical analyses were performed using IBM SPSS Statistics (version 19, SPSS Inc., Chicago, IL, USA).

## Results

### Patient characteristics

Ten patients (6 men, 4 women) with AS as classified by the modified New York criteria and a median age of 44 years (range 30 to 58 years) participated in this study. They had a median BASDAI of 4.9 (range 4.1 to 7.4) and a median Bath Ankylosing Spondylitis Functional Index (BASFI) of 4.6 (range 4.2 to 7.5). The median C-reactive protein level was 4.5 mg/l (range 1 to 41). Time interval between both imaging modalities ranged from 2 to 38 days (Table 1).

**Table 1:** Patient characteristics

**Patient**	**Gender**	**Age**	**BASDAI**	**BASFI**	**CRP**	**Time between PET/CT and MRI (days)**
1	m	56	4.3	4.2	2	9
2	w	38	4.7	4.8	17	29
3	m	47	4.1	4.3	11	2
4	w	55	7.4	6.8	1	16
5	w	30	5.1	4.4	6	3
6	m	43	7.1	7.5	2	7
7	m	58	4.7	4.4	41	3
8	w	44	5.4	6.0	1	21
9	m	44	4.1	4.2	3	38
10	m	32	6.8	6.4	24	18

*BASDAI,* Bath Ankylosing Spondylitis Disease Activity Index; *BASFI*, Bath Ankylosing Spondylitis Functional Index; *CRP*, C-reactive protein (mg/l); m, men; w, women.

### Interobserver agreement for F PET/CT and MRI of spinal lesions

Reader agreement was assessed by kappa statistics for each imaging modality. The interobserver agreement for F PET/CT was 0.68 for spinal lesions and 0.88 for SIJ lesions. Interobserver agreement for MRI ranged from 0.64 for spinal lesions to 0.93 for the SIJ lesions.

### Anatomic distribution of lesions detected by F PET/CT and MRI

All patients had increased radionuclide uptake in at least one area on F PET/CT (spine and SIJ in four patients, only in the spine in four patients, and only in the SIJ in two patients). A total of 38 areas with increased uptake in the spine were detected by F PET/CT: 17 vertebral body lesions (10 aCIL, 3 LIL, 4 NIL) and 21 lesions within the posterior spine structures, with the thoracic spine most frequently affected (30/38 = 79% of lesions). With regard to SIJ involvement, radionuclide uptake was recorded in 17 SIJ quadrants.

Nine out of ten patients had inflammatory lesions on MRI (four patients had lesions in both spine and SIJ, two patients had lesions only in the spine, and three patients had only SIJ lesions). MRI detected 68 inflammatory spinal lesions (41/68 = 60% of them in the thoracic spine), 52 vertebral body lesions (21 aCIL, 9 pCIL, 19 LIL, 3 NIL), and 16 posterior lesions. Nineteen SIJ quadrants showed signs of bone marrow edema (Tables 2 and 3).

**Table 2:** Distribution of spinal lesions on PET/CT and MRI

	**u-aCIL**	**u-pCIL**	**l-aCIL**	**l-pCIL**	**u-LIL ri**	**u-LIL le**	**I-LIL ri**	**I-LIL le**	**NIL**	**CPIL**	**PIL**	**FIL**
**Patient 1**												
PET/CT									C6		Th10	
MRI		L3		L1, L2	S1	S1	L5	L5				
**Patient 2**												
PET/CT												
MRI												
**Patient 3**												
PET/CT												
MRI												
**Patient 4**												
PET/CT									Th11			Th6, L3, S1
MRI												
**Patient 5**												
PET/CT	L2											
MRI	Th4											
**Patient 6**												
PET/CT											Th6	
MRI												
**Patient 7**												
PET/CT	Th2, Th3, Th 5-10					Th7			Th8, Th10		Th1-12	L1
MRI	Th1, Th3, Th8, Th10-12	Th5	Th2, Th8-12, L2	Th4	L1-3	Th3, Th7	L2, L3	Th3-5, L2	Th9-11		Th3-10, L1, L4	Th3-7, Th12
**Patient 8**												
PET/CT			C3				C5					Th5
MRI						L5						
**Patient 9**												
PET/CT							C5					
MRI	C7, Th1, L4, L5	C6, C7	C6	C5, C6	C6, Th1		C5					
**Patient 10**												
PET/CT											Th10	Th10
MRI	Th5		Th4									

a, anterior; CIL, corner inflammatory lesion; CPIL, cervical posterior inflammatory lesion; FIL, facet joint inflammatory lesion; l, lower; le, left; LIL, lateral inflammatory lesion; NIL, non-corner inflammatory lesion; p, posterior; PIL, posterior element inflammatory lesion; u, upper; ri, right.

**Table 3:** Distribution of lesions in sacroiliac joint quadrants on PET/CT and MRI

	**Positive sacroiliacal joint quadrants**
**Patient 1**	
PET/CT	
MRI	
**Patient 2**	
PET/CT	R-Inf-Iliac, R-Inf-Sacr, L-Inf-Sacr
MRI	R-Inf-Iliac, R-Inf-Sacr, L-Inf-Sacr
**Patient 3**	
PET/CT	R-Inf-Iliac, R-Inf-Sacr, L-Inf-Iliac, L-Inf-Sacr
MRI	R-Inf-Sacr, L-Inf-Sacr
**Patient 4**	
PET/CT	
MRI	L-Inf-Iliac
**Patient 5**	
PET/CT	R-Inf-Iliac, R-Inf-Sacr, L-Inf-Iliac, L-Inf-Sacr
MRI	R-Inf-Iliac, R-Sup-Sacr, R-Inf-Sacr, L-Inf-Iliac, L-Sup-Sacr, L-Inf-Sacr
**Patient 6**	
PET/CT	
MRI	
**Patient 7**	
PET/CT	R-Sup-Iliac, R-Inf-Iliac
MRI	
**Patient 8**	
PET/CT	R-Inf-Iliac, L-Inf-Iliac
MRI	R-Inf-Iliac,R-Inf-Sacr,L-Inf-Iliac,L-Inf-Sacr
**Patient 9**	
PET/CT	
MRI	L-Inf-Sacr
**Patient 10**	
PET/CT	L-Inf-Iliac,L-Inf-Sacr
MRI	L-Inf-Iliac,L-Inf-Sacr

*Inf*, inferior; *L*, left; *R*, right; *Sacr*, sacral; *Sup*, superior.

### Association between radionuclide uptake in F PET/CT and inflammation on MRI for the spine

**Figure 1:**
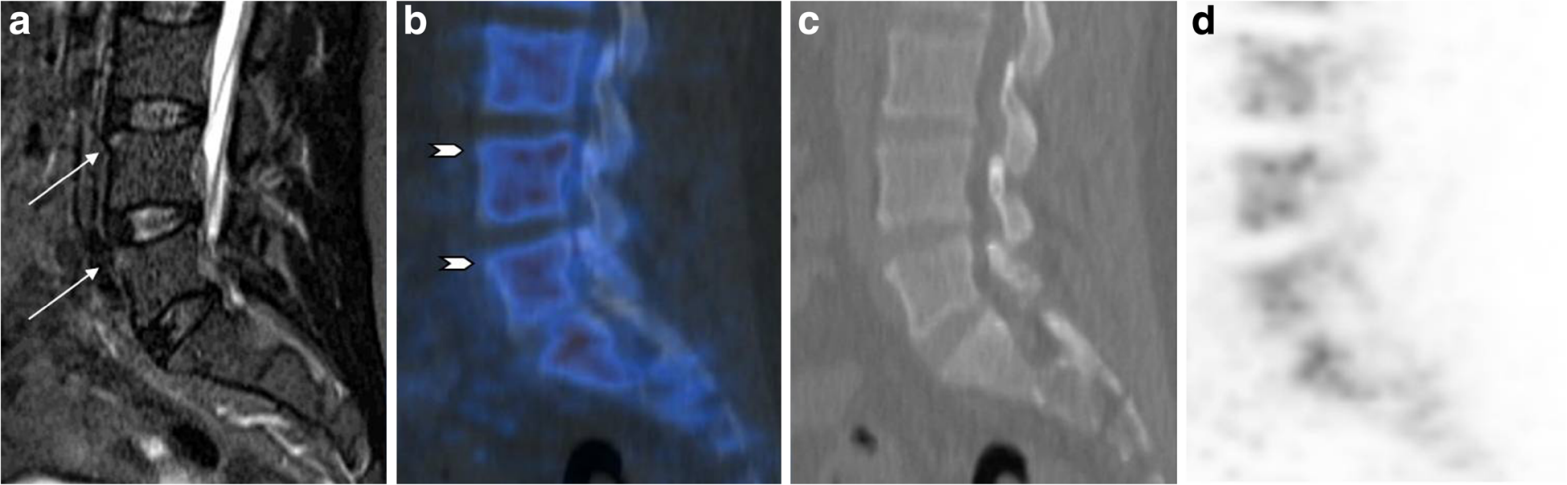
** Forty-four-year-old male patient (patient number 9).** Patient with an u-aCIL L4, L5 on MRI (arrows) without corresponding increased activity on F PET/CT (arrowheads). Sagittal STIR (**a**), fused PET/CT (**b**), CT (**c**), and PET (**d**) images.

**Figure 2:**
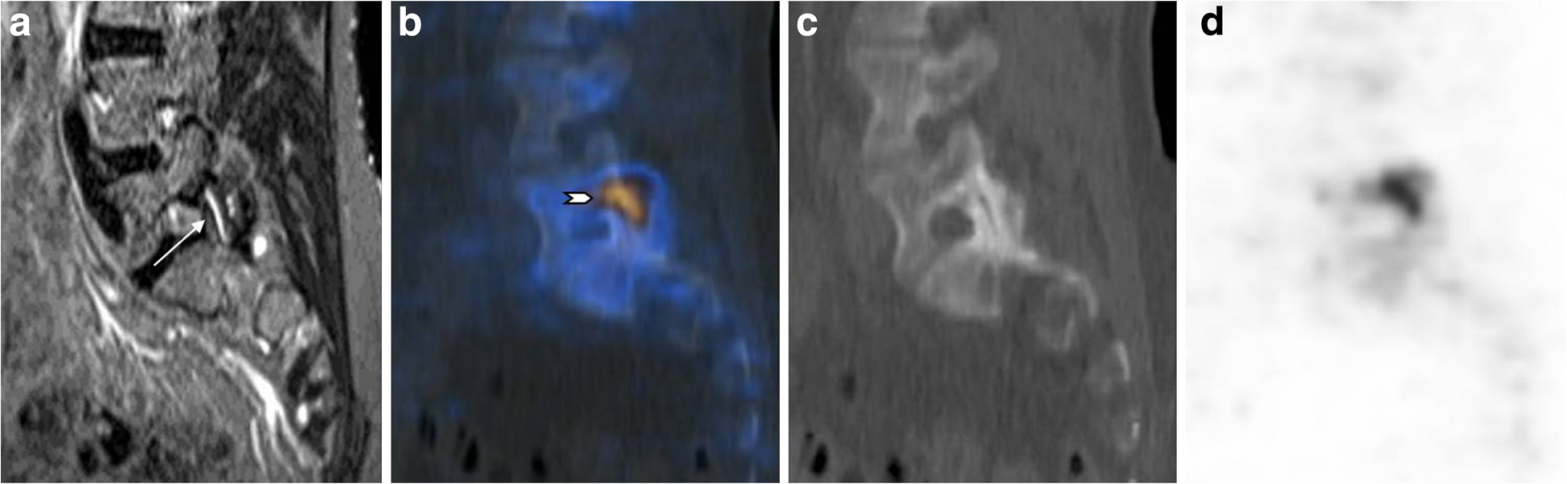
** Fifty-five-year-old female patient (patient number 4).** Patient with fluid within the left facet joint S1 (arrow) on MRI and corresponding increased activity on F PET/CT (arrowhead) indicating activated degenerative facet joint osteoarthritis. Left parasagittal STIR (**a**), fused PET/CT (**b**), CT (**c**), and PET (**d**) images.

Only 14 spine lesions were recorded concordantly by both methods (MRI and F PET/CT): 6 vertebral body lesions and 8 lesions within the posterior spine structures. Twenty-four spinal lesions detected by F PET/CT had no inflammatory character on MRI, while only a minority of them corresponded to fatty changes on T1-weighted MRI sequences (data not shown). A total of 54 acute inflammatory lesions on MRI showed no increased activity on F PET/CT imaging (Table 4). An example is shown in Figure 1. Analysis of consensus reading of PET/CT and MRI with regard to agreement of spinal lesion detection between both imaging modalities yielded a kappa value of only 0.25. Some lesions detected may be the consequence of concurrent degenerative spine disease as exemplified in Figure 2.

**Table 4:** Number of associated and non-associated positive lesions in PET/CT and/or MRI in consensus reading

	**PET/CT positive/MRI positive**	**PET/CT negative/MRI positive**	**PET/CT positive/MRI negative**
Spinal lesions	14	54	24
SIJ lesions	13	6	4

### Association between radionuclide uptake in F PET/CT and inflammation on MRI for SIJ quadrants

**Figure 3:**
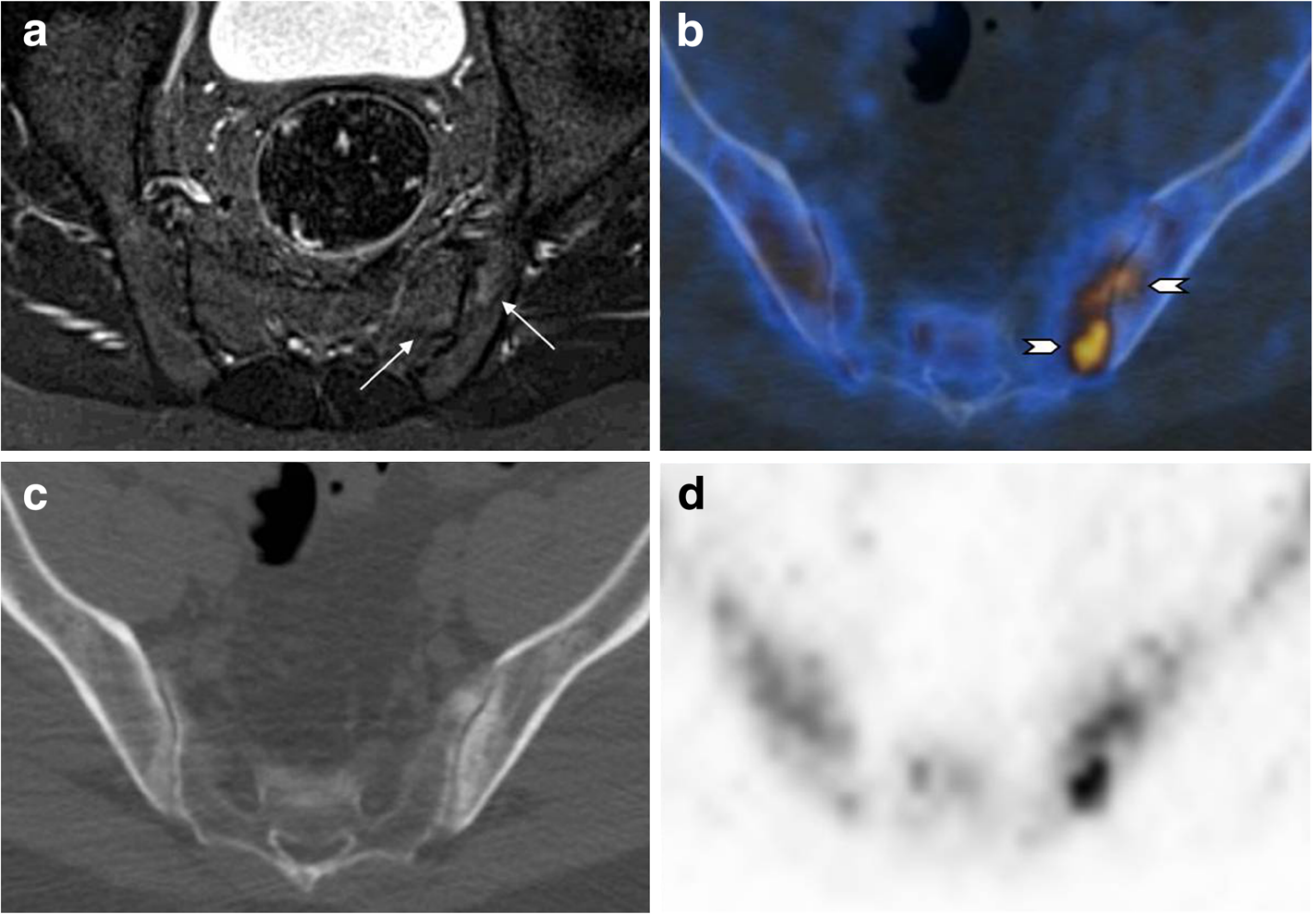
** Thirty-two-year-old male patient (patient number10).** Patient with positive findings in the inferior iliac and sacral quadrant of the left iliosacral joint both on MRI (edema, arrows) and on F PET/CT (arrowheads). Axial STIR (**a**), fused PET/CT (**b**), CT (**c**), and PET (**d**) images.

**Figure 4:**
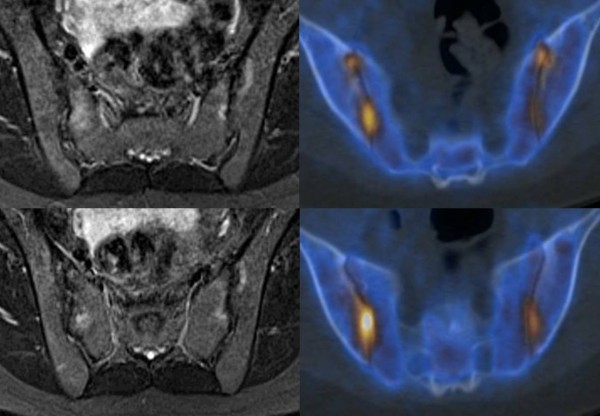
** Thirty-year-old female patient (patient number 5).** Patient with positive findings in all four inferior iliosacral quadrants both on MRI and F PET/CT without exact overlap. Axial STIR images (left) and fused PET/CT images (right).

A better agreement between the two imaging methods was found for inflammation in SIJ quadrants (kappa value 0.64): 13/80 SIJ quadrants had lesions recorded by both methods, while 4 lesions were detected only by F PET/CT, and 6 only by MRI (Table 4, Figure 3). However, the evaluated SIJ quadrants were quite large, and retrospective comparison of the changes of SIJ quadrants in both modalities showed that in the SIJ edema and increased 18 F-fluoride uptake, although situated in the same quadrants, did not all show an exact overlap (Figure 4).

## Discussion

The results of the present descriptive pilot study suggest that the radiotracer uptake detected by F PET/CT in the spine and SIJ of patients with AS is only marginally associated with the concomitant MRI evidence of inflammation. The relationship between active inflammatory MRI lesions at vertebral corners and the development of syndesmophytes as assessed on conventional radiographs has been analyzed in several recent studies [8-12,18,19]. Although clear longitudinal data are still lacking, these studies point to a process that comprises resolution of inflammation, activation of repair mechanisms involving fatty transformation of the lesion followed by new bone formation. However, new syndesmophytes may develop at sites without inflammation or fat infiltration on baseline MRI [11,12]. These findings suggest the presence of non-inflammatory pathways, although the presence of underlying inflammation not detectable by MRI cannot be formally excluded. F PET/CT might be able to detect the anabolic repair process leading to new bone formation regardless of the definite pathophysiological pathway. It was therefore anticipated that 18 F-fluoride uptake might also occur in active mineral deposition associated with degenerative spine disease (as exemplified in Figure 2), especially as patients with established, longstanding AS were included in this study. Longitudinal studies in patients with early axial SpA with only minimal degenerative disease are warranted.

Also, as this is a rather small study group (*n* = 10), further studies have to be performed with a larger patient population to verify our findings. As a next step, repeated imaging and clinical observations over a long time period would be necessary in order to appreciate the predictive value and the clinical relevance of the (discrepant) findings in both imaging modalities described, including their impact on patient management.

In this study, we focused on the functional part of F PET/CT. The low-dose CT part was used for attenuation correction and for lesion localization. Evaluation of the CT findings (especially if adding a dedicated thin-slice CT) and their potential contribution to the diagnostic process could be of additional value.

In contrast to spinal disease, a comparable frequency of increased 18 F-fluoride uptake on PET/CT and bone marrow edema on whole-body MRI was found at the level of SIJ quadrants, with a substantial agreement in their distribution between the two methods. Yet, within the same quadrant, increased radionuclide uptake and bone marrow edema did not all show an exact overlap.

## Conclusion

F PET/CT may provide additional information to MRI evaluation in patients with AS, both in spinal and in SIJ involvement. Whether radionuclide uptake detected with this method or with hybrid 18 F-fluoride PET/MRI technology performed on the same day might be able to better predict syndesmophyte formation, a hallmark of the disease, should be investigated in larger studies.

## Endnotes

This study was approved by the Institutional Review Board (IRB), and Swissmedic was duly notified. Amendments to the protocol for this study, suppressing bone scans, and extending the number of subjects were also approved by the IRB; however, it was omitted to notify Swissmedic about these amendments.

## Abbreviations

### Lab values

BASDAI: Bath Ankylosing Spondylitis Disease Activity Index; BASFI: Bath Ankylosing Spondylitis Functional Index; CRP: C-reactive protein (mg/l).; A: ANTERIOR; CIL: Corner inflammatory lesion; CPIL: Cervical posterior inflammatory lesion; FIL: Facet joint inflammatory lesion; L: Lower; LE: Left; LIL: Lateral inflammatory lesion; NIL: Non-corner inflammatory lesion; P: Posterior; PIL: Posterior element inflammatory lesion; U: Upper; ri: Right; Inf: Inferior; L: Left; R: Right; Sacr: Sacral; SIJ: Sacroiliac joint; Sup: Superior; AS: Ankylosing spondylitis; F-PET/CT: 18F-fluoride PET/CT; SpA: Spondyloarthritis.

### Spine lesions

BASDAI: Bath Ankylosing Spondylitis Disease Activity Index; BASFI: Bath Ankylosing Spondylitis Functional Index; CRP: C-reactive protein (mg/l).; A: ANTERIOR; CIL: Corner inflammatory lesion; CPIL: Cervical posterior inflammatory lesion; FIL: Facet joint inflammatory lesion; L: Lower; LE: Left; LIL: Lateral inflammatory lesion; NIL: Non-corner inflammatory lesion; P: Posterior; PIL: Posterior element inflammatory lesion; U: Upper; ri: Right; Inf: Inferior; L: Left; R: Right; Sacr: Sacral; SIJ: Sacroiliac joint; Sup: Superior; AS: Ankylosing spondylitis; F-PET/CT: 18F-fluoride PET/CT; SpA: Spondyloarthritis.

### Sacroiliac joint lesions

BASDAI: Bath Ankylosing Spondylitis Disease Activity Index; BASFI: Bath Ankylosing Spondylitis Functional Index; CRP: C-reactive protein (mg/l).; A: ANTERIOR; CIL: Corner inflammatory lesion; CPIL: Cervical posterior inflammatory lesion; FIL: Facet joint inflammatory lesion; L: Lower; LE: Left; LIL: Lateral inflammatory lesion; NIL: Non-corner inflammatory lesion; P: Posterior; PIL: Posterior element inflammatory lesion; U: Upper; ri: Right; Inf: Inferior; L: Left; R: Right; Sacr: Sacral; SIJ: Sacroiliac joint; Sup: Superior; AS: Ankylosing spondylitis; F-PET/CT: 18F-fluoride PET/CT; SpA: Spondyloarthritis.

### Miscellaneous

BASDAI: Bath Ankylosing Spondylitis Disease Activity Index; BASFI: Bath Ankylosing Spondylitis Functional Index; CRP: C-reactive protein (mg/l).; A: ANTERIOR; CIL: Corner inflammatory lesion; CPIL: Cervical posterior inflammatory lesion; FIL: Facet joint inflammatory lesion; L: Lower; LE: Left; LIL: Lateral inflammatory lesion; NIL: Non-corner inflammatory lesion; P: Posterior; PIL: Posterior element inflammatory lesion; U: Upper; ri: Right; Inf: Inferior; L: Left; R: Right; Sacr: Sacral; SIJ: Sacroiliac joint; Sup: Superior; AS: Ankylosing spondylitis; F-PET/CT: 18F-fluoride PET/CT; SpA: Spondyloarthritis.

## Competing interests

GvS is a consultant to Icon Medical Imaging plc and grant recipient from GE Healthcare. All other authors declare that they have no competing interests.

## Authors’ contributions

DRF conceived and designed the study, read out, acquired, analysed, and interpreted the data, and drafted, wrote, and revised the manuscript. CWAP conceived and designed, read out, and revised the manuscript. VZ read out, wrote, and revised the manuscript. KDMS read out and revised the manuscript. BS has done the statistics and revised the manuscript. KS acquired the IRB approval, recruited patients, and revised the manuscript. GT recruited patients and revised the manuscript. GvS acquired the IRB approval and revised the manuscript. BAM recruited patients and revised the manuscript. AC conceived and designed the study, analysed and interpreted the data, drafted, wrote, and revised the manuscript, is the guarantor of the integrity of the study, and approved the final version to be published. All authors read and approved the final manuscript.
